# Nurses' Food

**Published:** 1886-10-16

**Authors:** 


### NURSES' FOOD.

Being much interested in the subject of hospital nurses' food, may I offer a few suggestions in addition to those published by you in your first issue of The Hospital? My experience is that hospital nurses are a very difficult body to feed. The great pressure of their work gives them but little time for their meals; the immediate return to their work after their meals does not assist their digestion; and the very nature of them takes off the edge of their appetite. Another difficulty is, though easily overcome, that, as a rule, hospitals provide plenty of meat for the nurses' table, but meat only. Now, nurses do not take to lumps of meat. How often have I gone to my dinner, perhaps after a seven hours' fast, hard at work all the time, with a sinking, aching void within me, but the lumps were too much for me. I had got my heart's desire, food, but barren withal. Many times I have left my dinner untasted, and returned to my ward with perhaps a cup of tea to sustain me from six in the morning to six at night; and then I had some more tea. Nurses work hard, and their work is often revolting—their meals should therefore be made appetising, varied, and be also nicely served. The fact of knowing what your dinner is to be beforehand takes away most of its enjoyment.

I should suggest therefore that there should be more puddings on the diet-list—more vegetables—soup twice a week for supper, and cold meat with salad, which is greatly appreciated. With a very little trouble, an extensive diet-list could be arranged, say a three weeks' list instead of one; it would not cost the hospital much more, and the authorities would have the satisfaction of seeing their nurses doing their daily work with more health and in better spirits. I do think that those who labour so arduously for their fellow creatures should have their own personal comforts a little more considered. I enclose a *menu* for three weeks.

Victoria.

*Sunday.*—Cold Pork, Salad, Tart.
Monday.—Boiled Mutton, Caper Sauce, two Vegetables.
Tuesday.—Boiled Beef, Rice Pudding.
Wednesday.—Cold Roast Mutton, Salad, Jam Pudding.
Thursday.—Beef Steak Pudding, two Vegetables.
Friday.—Fish, Boiled Raisin Pudding.
Saturday.—Irish Stew, two Vegetables.
*Sunday.*—Cold Veal, Salad, Tart.
Monday.—Roast Mutton, browned Potatoes.
Tuesday.—Stewed Steak, Sago Pudding.
Wednesday.—Cold Meat, Beet-root, Currant Dumpling.
Thursday.—Sausages, Mashed Potatoes.
Friday.—Fish, Boiled Fruit Pudding.
Saturday.—Minced Fresh Meat, with Potato Crust.
*Sunday.*—Cold Beef, Celery, Tart.
Monday.—Meat Pies, two Vegetables.
Tuesday.—Roast Beef, light Pudding.
Wednesday.—Cold Meat, Salad, Suet Pudding and Treacle.
Thursday.—Stewed Rabbits, two Vegetables.
Friday.—Fish, Boiled Raisin Pudding.
Saturday.—Liver and Bacon, two Vegetables.

